# Spiders of the Udmurt Republic, Russia

**DOI:** 10.3897/BDJ.9.e70534

**Published:** 2021-08-24

**Authors:** Artëm Sozontov

**Affiliations:** 1 Institute of Plant and Animal Ecology (IPAE UB RAS), Ekaterinburg, Russia Institute of Plant and Animal Ecology (IPAE UB RAS) Ekaterinburg Russia

**Keywords:** Araneae, Aranei, abundance, diversity, life stage, occurrence, southern taiga, specimen, Udmurtia

## Abstract

**Background:**

The long-term project "Spiders of the Udmurt Republic" (2007–2018) aimed to research spiders' regional fauna and zoogeography, diversity (including spatial and seasonal patterns) and habitat preferences. We performed the collection of spiders in all natural zones of the republic, habitats and vegetation layers, both at permanent sampling plots and through ad-hoc sampling en route.

**New information:**

The dataset includes occurrences from 53 geographical points with 10,500 records and more than 35,000 specimens. This increases the existing data on Russian spiders on GBIF by four times, from 11,000 (excluding iNaturalist observations) to 46,000. The dataset allows for the exploration of regional fauna, local and general species distribution, spider phenology and habitat preferences for the purposes of monitoring and conservation.

## Introduction

L.K. Krulikovskiy provided the first data related to spiders of Udmurt Republic (Krulikovskiy 1892, Krulikovskiy 1908, Krulikovskiy 1915). A long time later, V.P. Tyshchenko noted ten spider species occurring in the Udmurt Republic in his book (Tyshchenko 1971). T.L. Zubko carried out the first detailed faunistic research on the spiders of the Republic between 1978 and 1981 where she recorded 72 spider species (Zubko and Roshchinenko 1981), but her collection is currently lost.

In 2007, I started studying the spiders of Udmurtia with a focus on their fauna, diversity, biogeography, ecology and community structure. The study was conducted in cooperation with Prof. S.L. Esyunin (Perm State University) and Prof. S.V. Dedyukhin (Udmurt State University). The first published article (Sozontov and Esyunin 2012) increased the number of known species by 2.5 times (Table 1). In 2013, the entire research history was reviewed to summarise all published data and outline prospects of future investigations (Sozontov 2013). Following this, a number of articles about the region's fauna, biogeography and taxonomy were published (Adakhovskiy et al. 2012, Sozontov and Esyunin 2015, Esyunin and Sozontov 2015, Sozontov 2015a). Finally, the manuscript of my own PhD thesis "Fauna and ecology of spiders (Aranei) of the Udmurt Republic: diversity, habitat distribution, community structure" contains an integrated analysis of all the data concerning spider fauna structure and origins, spider community diversity and structure and their spatiotemporal variation (Sozontov 2018).

**Figure 1.**
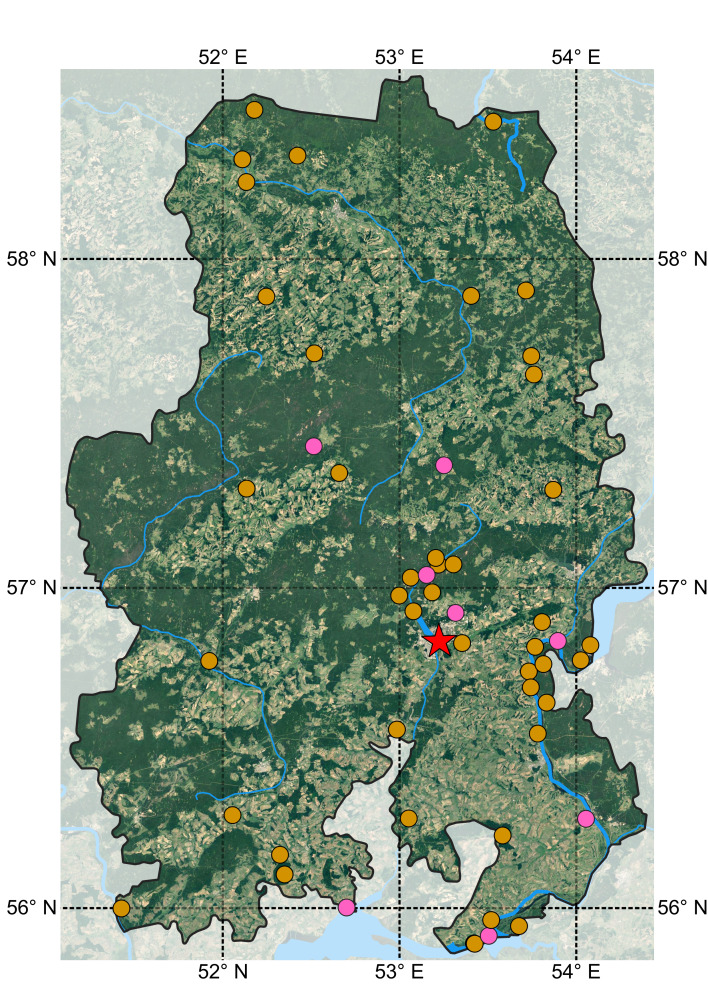
The studied region and localities of collection (all symbols). Purple circles show well-studied local faunas. The red star shows Izhevsk, the capital of the Udmurt Republic.

This research project was conducted according to the principles and methods of ecological-faunistic investigations (Dedyukhin 2011). The main idea of this approach is combining detailed study of reference local faunas (Chernov and Penev 1993, Penev 1996, Penev 1997) and ad-hoc sampling en route. The ecological-faunistic approach assumes sampling in all natural zones of the Republic, habitats and vegetation layers (from soil litter to canopy) with relevant methods. Thus, we collected quantitative data on eight local faunas and species occurrences at 45 occasional points, which are helpful for exploring the regional distribution of species and maximising the proportion of discovered fauna. Well-studied local faunas for the project are marked in purple on the map shown in Fig. 1 and include the "Siva" permanent field study station, Ust-Belsk, Golushurma, Sokolovka, "Sirius" SNT, Novye Zyatci, Chutyr and Hohryaki. Table 2 contains their coordinates and amount of material collected.

## Project description

### Title

Spiders of the Udmurt Republic

### Personnel

Artëm Sozontov

## Sampling methods

### Study extent

**Figure 2.**
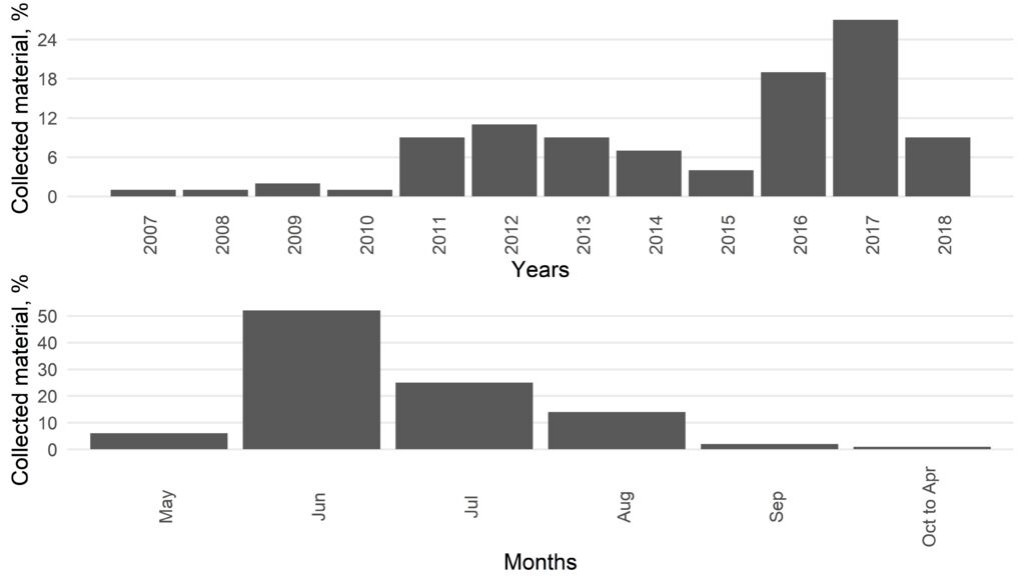
Distribution of collected material across years and months.

The dataset includes spider (order Araneae) species occurrences within administrative borders of the Udmurt Republic. The collection contains material picked up over 12 years, from 2007 to 2018. Occurrences presented in the dataset come from 53 geographical points providing around 10,500 records in the dataset with more than 35,000 specimens recorded (Sozontov 2021). This amount of data extends the current presence of Russian spiders on GBIF by approximately four times, from 11,000 (excluding iNaturalist observations) to 46,000 (Gbif.Org 2020). Typical seasonal coverage is from May to September, with a few exceptions. The total ratio of spiders by years and months is provided in Fig. 2. There is a total of 53 observed sampling places, collapsing to 21 types of biotopes: floodplain oak forests, riverine deciduous forest strips, watershed lime-forests, pine forests, spruce-fir forests, dark coniferous forests with lime, floodplain stepped meadows, bottomland meadows, upland meadows, sloping stepped meadows, edges of deciduous forests, edges of mixed forests, edges of pine-forests, open raised bogs, forested raised bogs, open lake/pond shores, open river banks, alder groves, buildings (heated and unheated) and agrocoenoses (agricultural fields, orchards, flower and kitchen gardens).

### Sampling description

Field studies were carried out using common methods: lines of pitfall traps, entomological nets, litter sifting and manual collecting (Tyshchenko 1971, Woodcock 2005, Oliger 2010). Plastic cups with a 7 cm diameter served as pitfall traps and 4% acetic acid with a pellet of surface-active substance as killing and preserving agents. Trap lines included 5 to 15 traps, with a spacing of 3 m. Spiders from grass, shrub and canopy (lower than 3 m) layers were collected using an entomological net of 35 cm hoop diameter and 150 cm handle length. All the pitfall traps and some of the entomological net-sweeping samples are quantitative and can be normalised to 100 trap-days or 200 sweeps. The litter sifting and manual collecting samples are qualitative.

### Quality control

All collected spider specimens were wet-preserved in 70% alcohol. Stored material is shared between the Institute of Plant and Animal Ecology (IPAE UB RAS, Ekaterinburg, the vast majority), Perm State University (Perm), Udmurt State University (Izhevsk) and the Zoological Museum of the Moscow State University (Moscow). Aside from our own material, the dataset involves the purposeful collections of Svetlana Shirobokova, Konstantin Tatarkin, Evgenia Shirobokova, Alesya Uskova and Sergey Dedyukhin and specimens occasionally collected by other colleagues, listed as legitimate collectors in the "recordedBy" DarwinCore column. The majority of the material has identification to species level (71%) and almost all the remaining to genus level (29%, primarily juveniles). Species identification was performed mainly by the author; about 10% of specimens were cross-checked or identified by Prof. S.L. Esyunin (Perm). Taxonomy nomenclature complies with the World Spider Catalog (World Spider Catalog 2021).

### Step description

The dataset is not a part of any ongoing projects.

## Geographic coverage

### Description

**Figure 3.**
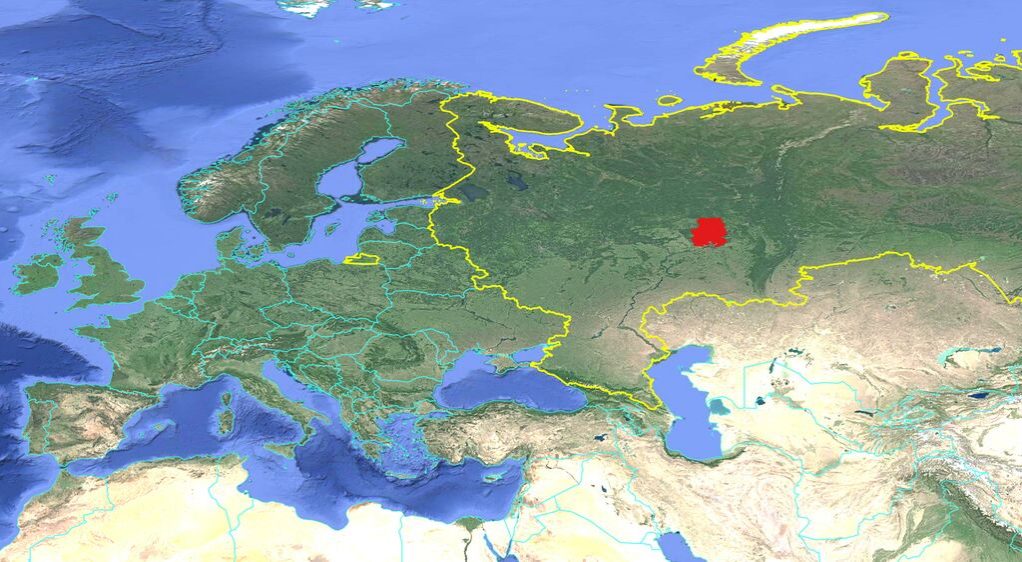
The position of the Udmurt Republic (red area) relatively to Russia (yellow borders) and other countries (cyan borders).

All collecting points are limited by administrative borders of the Udmurt Republic, one of the federal subjects of the Russian Federation (Fig. 3). Being 42,000 km^2^ in area, it extends 300 km in a north-south direction and 200 km in a west-east direction. The studied region belongs to the east of the Russian plain, borders with the western piedmont of the Ural Mountains and is situated in the interstream area of the Vyatka and Kama Rivers (Illarionov 2009). Relief of the Republic can be defined as a hilly plain. Regional altitudes lie within 52 and 333 m a.s.l. (Podsosova 1972); collecting points lie between 61 and 254 m a.s.l. The territory includes southern taiga (1/3) and mixed forest (2/3) subzones; the southernmost area is under the intense influence of the forest-steppe (Shadrin 1999, Baranova et al. 2010), which reflects on spider fauna notably.

### Coordinates

55.857 and 58.557 Latitude; 51.129 and 54.471 Longitude.

## Taxonomic coverage

### Description

The dataset includes 403 identified spider species of 181 genera and 26 families. About 25,000 individuals are identified to the species level (23,645 adult and 1,296 juvenile specimens) and about 10,000 individuals to the genus level (305 adult and 9678 juvenile specimens). Amongst them, there are 321 individuals with unclear species status and requiring further taxonomic investigations, belonging to Linyphiidae (189), Clubionidae (38), Thomisidae (24), Lycosidae (20), Theridiidae (13), Titanoecidae (9), Gnaphosidae (5), Tetragnathidae (5), Araneidae (5), Philodromidae (5), Dictynidae (3), Salticidae (2), Hahniidae (1), Cheiracanthiidae (1) and Mimetidae (1). Some of the linyphiids (16 adult and 223 juvenile specimens) remain identified only at the family level.

### Taxa included

**Table:** 

Rank	Scientific Name	Common Name
order	Araneae Clerck, 1757	Spiders

## Temporal coverage

### Notes

2007-06-21 through 2018-08-02

## Usage licence

### Usage licence

Other

### IP rights notes

Creative Commons Attribution (CC-BY) 4.0 License.

## Data resources

### Data package title

Spiders of the Udmurt Republic, Russia

### Resource link

https://www.gbif.org/dataset/01f33154-d6f7-4079-a9e2-f0a9ca8e8630

### Alternative identifiers

https://doi.org/10.15468/nvzbxq, http://gbif.ru:8080/ipt/resource?r=spiders_udm21

### Number of data sets

1

### Data set 1.

#### Data set name

Spiders of the Udmurt Republic, Russia

#### Data format

Darwin Core

#### Number of columns

57

#### Description

The dataset is based on the results of a long-term project, "Spiders of the Udmurt Republic" (2007–2018), with some additional data. Samples come from 53 geographical points providing about 10,500 records in the dataset with more than 35,000 specimens recorded. There are 403 spider species in the studied fauna, of 181 genus and 26 families (Table 3). The collected specimens have the age-sex distribution: males (40%), females (28%), subadult spiders (11%) and juveniles (21%). The dataset can help to explore regional fauna, regional and general species distribution, their monitoring, conservation, phenology and relationships with biotic and abiotic conditions.

**Data set 1.** 

Column label	Column description
type	The nature or genre of the resource. A variable ("PhysicalObject" for preserved specimen, "Event" for observed or missed individuals).
modified	The most recent date-time on which the resource was changed. A constant ("YYYY-MM-DD").
language	A language of the resource. A constant ("en" = English).
licence	A legal document giving official permission to do something with the resource. A constant ("CC_BY_4_0" = Creative Commons Attribution (CC-BY) 4.0 Licence).
rightsHolder	A person or organisation owning or managing rights over the resource. A constant ("Institute of Plant and Animal Ecology (IPAE), UB RAS").
bibliographicCitation	A bibliographic reference for the resource as a statement indicating how this record should be cited (attributed) when used. A variable.
institutionCode	The name (or acronym) in use by the institution having custody of the object(s) or information referred to in the record. A constant ("Institute of Plant and Animal Ecology (IPAE), UB RAS").
datasetName	The name identifying the dataset from which the record was derived. A constant ("Spiders of the Udmurt Republic").
basisOfRecord	The specific nature of the data record. A variable ("PhysicalObject" for preserved specimen, "Event" for observed or missed individuals).
occurrenceID	An identifier for the occurrence. A variable, constructed from a combination of Protected Area's name (in case of location belongs to one of them) or "id" and row number.
catalogNumber	An identifier (preferably unique) for the record within the dataset or collection. A variable.
recordedBy	A list (concatenated and separated) of names of people, groups or organisations responsible for recording the original occurrence. A variable.
individualCount	The number of individuals represented present at the time of the Occurrence. A variable.
sex	The sex of the biological individual(s) represented in the Occurrence (males, females and juvenile specimens counted, concatenated and separated by "," in case of mix sex-age stages in a sample). A variable.
lifeStage	The life stage of the biological individual(s) at the time the Occurrence was recorded. A variable (three terms: "adult", "juvenile" or "adult, juvenile").
occurrenceStatus	A statement about the presence or absence of a taxon at a location. A constant ("present").
disposition	The current state of a specimen with respect to the collection identified in collectionCode or collectionID. A variable (three terms: "in collection", "missing", "duplicates elsewhere" and "voucher elsewhere").
associatedReferences	A list (concatenated and separated) of bibliographic references of literature associated with the Occurrence. A variable.
associatedTaxa	A list (concatenated and separated) of identifiers or names of taxa and their associations with the Occurrence. A variable (edificator plant species for forest habitats).
fieldNumber	An identifier given to the event in the field. A variable.
eventDate	The date-time or interval during which an Event occurred. For occurrences, this is the date-time when the event was recorded. A variable.
startDayOfYear	The earliest integer day of the year on which the Event occurred. A variable.
endDayOfYear	The latest integer day of the year on which the Event occurred. A variable.
year	The four-digit year in which the event occurred, according to the Common Era Calendar. A variable.
month	The ordinal month in which the event occurred. A variable.
day	The integer day of the month on which the event occurred. A variable.
verbatimEventDate	The verbatim original representation of the date and time information for an Event. A variable.
habitat	A category of the habitat in which the Event occurred. A variable.
locationID	An identifier for the place of field data collection. A variable.
higherGeography	A list (concatenated and separated) of geographic names less specific than the information captured in the locality term. A variable with the constant begin ("Europe | Russia | Udmurt Republic").
continent	The name of the continent in which the location occurs. A constant ("Europe").
country	The name of the country or major administrative unit in which the location occurs. A constant ("Russia").
countryCode	The standard code for the country in which the location occurs. A constant ("RU").
stateProvince	The name of the next smaller administrative region than country (state, province, canton, department, region etc.) in which the location occurs. A constant ("Udmurt Republic").
county	The full, unabbreviated name of the next smaller administrative region than stateProvince in which the Location occurs. A variable.
locality	The specific description of the place. Less specific geographic information is provided in other geographic terms (higherGeography, continent, country, stateProvince, county). This term may contain information modified from the original to correct perceived errors or standardise the description. A variable.
verbatimLocality	The original textual description of the place. A variable.
minimumElevationInMetres	The lower limit of the range of elevation (altitude above sea level), in metres. A variable.
maximumElevationInMetres	The upper limit of the range of elevation (altitude above sea level), in metres. A variable.
locationRemarks	Comments or notes about the Location. A constant ("Vyatka & Kama interstream area | the Cis-Ural region | western piedmont of the Ural Mountains | the east of the Russian Plain").
decimalLatitude	The geographic latitude (in decimal degrees, using the spatial reference system given in geodeticDatum) of the geographic centre of a location. A variable.
decimalLongitude	The geographic longitude (in decimal degrees, using the spatial reference system given in geodeticDatum) of the geographic centre of a location. A variable.
geodeticDatum	The ellipsoid, geodetic datum or spatial reference system (SRS) upon which the geographic coordinates given in decimalLatitude and decimalLongitude are based. A constant ("WGS84").
coordinateUncertaintyInMetres	The horizontal distance (in metres) from the given decimalLatitude and decimalLongitude describing the smallest circle containing the whole of the location. A variable.
georeferencedBy	A list (concatenated and separated) of names of people, groups or organisations who determined the georeference (spatial representation) of the location. A constant ("Sozontov A.N.").
georeferencedDate	The date on which the Location was georeferenced. A variable.
identifiedBy	A list (concatenated and separated) of names of people, groups, or organisations who assigned the Taxon to the subject. A variable.
dateIdentified	The date on which the subject was determined as representing the Taxon. A variable (YYYY-MM-DD).
identificationRemarks	Comments or notes about the Identification. A variable.
scientificName	The full scientific name, with authorship and date information. A variable.
acceptedNameUsage	The full name, with authorship and date information, if known, of the currently valid zoological taxon. A variable.
order	The full scientific name of the order in which the taxon is classified. A constant ("Araneae").
family	The full scientific name of the family in which the taxon is classified. A variable.
genus	The full scientific name of the genus in which the taxon is classified. A variable.
specificEpithet	The name of the species epithet of the scientificName. A variable.
scientificNameAuthorship	The authorship information for the scientificName, formatted according to the conventions of the applicable nomenclaturalCode. A variable.
taxonRank	The taxonomic rank of the most specific name in the scientificName. A variable (three options: "SPECIES", "GENUS" and "FAMILY").

## Additional information

Sozontov A (2021). Spiders of the Udmurt Republic, Russia. Version 1.3. Institute of Plant and Animal Ecology (IPAE). Occurrence dataset https://doi.org/10.15468/nvzbxq accessed via GBIF.org on 2021-05-28.

## Figures and Tables

**Table 1.** Spiders' faunistic data accumulation within the Udmurt Republic.

**Reference**	**Number of spider species**		
	**recorded** **in the paper**	**new to the** **regional fauna**	**total** **in the region**
Krulikovskiy 1892	1	1	1
Krulikovskiy 1908	4	3	4
Krulikovskiy 1915	1	0	4
Tyshchenko 1971	10	10	14
Zubko and Roshchinenko 1981	72	66	80
Zubko 2001	3	1	81
Sozontov and Esyunin 2012	195	122	203
Sozontov 2012a	1	1	204
Sozontov 2012b	17	5	209
Adakhovskiy et al. 2012	2	0	209
Sozontov and Esyunin 2014	158	47	256
Sozontov and Shirobokova 2014	40	40	296
Ermolaev et al. 2014	26	2	298
Sozontov and Esyunin 2015	2	0	298
Esyunin and Sozontov 2015	1	0	298
Sozontov 2015a	18	4	302
Sozontov 2015b	34	9	311
Sozontov 2018 (manuscript)	402	91	402
Sozontov 2021	403	1	403

**Table 2.** Well-studied local faunas

Name	Coordinates	Samples	Specimens	Observed habitats
"Siva" permanent field study station	56.827 53.905	912	2,888	Bottomland meadows, dark coniferous forests with lime, edges of deciduous forests, floodplain oak forests, floodplain steppificated meadows, open lake/pond shores, unheated buildings, upland meadows, watershed lime-forests, pine forests, sloping stepped meadows
Ust-Belsk	55.898 53.484	581	1,791	Agrocoenoses, floodplain oak forests, floodplain stepped meadows, heated buildings, open lake/pond shores, open river banks, pine forests, riverine deciduous forest strips, sloping stepped meadows, spruce-fir forests, unheated buildings
Golushurma	56.002 52.701	457	1,952	Edges of deciduous forests, open lake/pond shores, riverine deciduous forest strips, sloping stepped meadows, spruce-fir forests, upland meadows
Sokolovka	56.281 54.054	544	7,492	Bottomland meadows, edges of mixed forests, floodplain oak forests, floodplain stepped meadows, sloping stepped meadows, upland meadows
"Sirius" SNT	57.033 53.144	826	3,047	Dark coniferous forests with lime, pine forests
Novye Zyatci	57.434 52.515	147	575	Dark coniferous forests with lime, edges of pine-forests, open raised bogs, pine forests
Chutyr	57.376 53.252	828	2,447	Agrocoenoses, alder groves, bottomland meadows, dark coniferous forests with lime, edges of mixed forests, edges of pine-forests, floodplain stepped meadows, open raised bogs, open river banks, sloping stepped meadows, spruce-fir forests, unheated buildings, upland meadows
Hohryaki	56.923 53.317	123	773	Dark coniferous forests with lime, heated buildings, watershed lime-forests

**Table 3.** The list of the spiders of the Udmurt Republic with the number of collected specimens and the number of localities and types of habitats where a species occurs.

**Family**	**Species**	**Individuals**	**Localities**	**Habitats**
Agelenidae	*Agelenalabyrinthica* (Clerck, 1758)	1	1	1
Agelenidae	*Tegenariadomestica* (Clerck, 1758)	43	5	4
Anyphaenidae	*Anyphaenaaccentuata* (Walckenaer, 1802)	6	3	2
Araneidae	*Aculepeiraceropegia* (Walckenaer, 1802)	33	10	10
Araneidae	*Agalenatearedii* (Scopoli, 1763)	125	10	9
Araneidae	*Araneusalsine* (Walckenaer, 1802)	6	5	5
Araneidae	*Araneusangulatus* Clerck, 1758	29	12	10
Araneidae	*Araneusdiadematus* Clerck, 1758	49	16	15
Araneidae	*Araneusmarmoreus* Clerck, 1758	42	18	14
Araneidae	*Araneusnordmanni* (Thorell, 1870)	1	1	1
Araneidae	*Araneusquadratus* Clerck, 1758	80	11	13
Araneidae	*Araneussaevus* (L. Koch, 1872)	1	1	1
Araneidae	*Araneussturmi* (Hahn, 1831)	8	3	7
Araneidae	*Araniellaproxima* (Kulczyński, 1885)	15	10	7
Araneidae	*Argiopebruennichi* (Scopoli, 1772)	14	5	5
Araneidae	*Cercidiaprominens* (Westring, 1851)	30	10	8
Araneidae	*Cyclosaconica* (Pallas, 1772)	29	11	7
Araneidae	*Cyclosaoculata* (Walckenaer, 1802)	3	3	3
Araneidae	*Hypsosingaheri* (Hahn, 1831)	10	5	5
Araneidae	*Hypsosingapygmaea* (Sundevall, 1831)	39	9	9
Araneidae	*Hypsosingasanguinea* (C. L. Koch, 1844)	7	5	5
Araneidae	*Larinioidescornutus* (Clerck, 1758)	53	13	11
Araneidae	*Larinioidesixobolus* (Thorell, 1873)	26	9	8
Araneidae	*Larinioidespatagiatus* (Clerck, 1758)	72	18	16
Araneidae	*Larinioidessclopetarius* (Clerck, 1758)	1	1	1
Araneidae	*Larinioidessuspicax* (O. Pickard-Cambridge, 1876)	47	7	10
Araneidae	*Leviellusstroemi* (Thorell, 1870)	31	6	5
Araneidae	*Mangoraacalypha* (Walckenaer, 1802)	124	19	19
Araneidae	*Neosconaadianta* (Walckenaer, 1802)	11	5	5
Araneidae	*Nucteneasilvicultrix* (C. L. Koch, 1835)	1	1	1
Araneidae	*Singahamata* (Clerck, 1758)	137	28	19
Araneidae	*Singanitidula* C. L. Koch, 1844	24	12	8
Cheiracanthiidae	*Cheiracanthiumerraticum* (Walckenaer, 1802)	93	24	18
Cheiracanthiidae	*Cheiracanthiumoncognathum* Thorell, 1871	1	1	1
Cheiracanthiidae	*Cheiracanthiumpennyi* O. Pickard-Cambridge, 1873	5	4	3
Clubionidae	*Clubionacaerulescens* L. Koch, 1867	13	6	7
Clubionidae	*Clubionadiversa* O. Pickard-Cambridge, 1862	2	2	2
Clubionidae	*Clubionafrisia* Wunderlich & Schuett, 1995	1	1	1
Clubionidae	*Clubionagermanica* Thorell, 1871	2	2	2
Clubionidae	*Clubionalutescens* Westring, 1851	10	7	6
Clubionidae	*Clubionaneglecta* O. Pickard-Cambridge, 1862	13	11	8
Clubionidae	*Clubionapallidula* (Clerck, 1758)	10	5	6
Clubionidae	*Clubionaphragmitis* C. L. Koch, 1843	12	4	2
Clubionidae	*Clubionapseudoneglecta* Wunderlich, 1994	2	2	1
Clubionidae	*Clubionareclusa* O. Pickard-Cambridge, 1863	10	5	5
Clubionidae	*Clubionasimilis* L. Koch, 1867	1	1	1
Clubionidae	*Clubionastagnatilis* Kulczyński, 1897	14	7	7
Clubionidae	*Clubionasubsultans* Thorell, 1875	3	2	2
Dictynidae	*Argennapatula* (Simon, 1874)	2	2	2
Dictynidae	*Argennasubnigra* (O. Pickard-Cambridge, 1861)	17	8	8
Dictynidae	*Argyronetaaquatica* (Clerck, 1758)	5	5	4
Dictynidae	*Brigittealatens* (Fabricius, 1775)	1	1	1
Dictynidae	*Dictynaarundinacea* (Linnaeus, 1758)	344	23	21
Dictynidae	*Dictynamajor* Menge, 1869	2	1	1
Dictynidae	*Dictynapusilla* Thorell, 1856	13	7	9
Dictynidae	*Dictynauncinata* Thorell, 1856	54	11	11
Dictynidae	*Emblynamitis* (Thorell, 1875)	1	1	1
Dictynidae	*Hackmaniaprominula* (Tullgren, 1948)	5	4	4
Dictynidae	*Lathysheterophthalma* Kulczyński, 1891	9	3	2
Dictynidae	*Lathyshumilis* (Blackwall, 1855)	3	1	1
Gnaphosidae	*Berlandinacinerea* (Menge, 1872)	1	1	1
Gnaphosidae	*Callilepisnocturna* (Linnaeus, 1758)	21	11	8
Gnaphosidae	*Drassodespubescens* (Thorell, 1856)	22	8	7
Gnaphosidae	*Drassodesvillosus* (Thorell, 1856)	8	5	4
Gnaphosidae	*Drassylluslutetianus* (L. Koch, 1866)	222	15	18
Gnaphosidae	*Drassylluspraeficus* (L. Koch, 1866)	54	12	11
Gnaphosidae	*Drassylluspusillus* (C. L. Koch, 1833)	150	14	14
Gnaphosidae	*Gnaphosalugubris* (C. L. Koch, 1839)	5	1	1
Gnaphosidae	*Gnaphosamontana* (L. Koch, 1866)	10	6	5
Gnaphosidae	*Haplodrassuscognatus* (Westring, 1861)	9	6	6
Gnaphosidae	*Haplodrassusmoderatus* (Kulczyński, 1897)	4	2	2
Gnaphosidae	*Haplodrassuspseudosignifer* Marusik, Hippa & Koponen, 1996	332	11	14
Gnaphosidae	*Haplodrassussignifer* (C. L. Koch, 1839)	45	9	8
Gnaphosidae	*Haplodrassussilvestris* (Blackwall, 1833)	104	10	6
Gnaphosidae	*Haplodrassussoerenseni* (Strand, 1900)	210	9	11
Gnaphosidae	*Haplodrassusumbratilis* (L. Koch, 1866)	402	12	13
Gnaphosidae	*Micariaaenea* Thorell, 1871	1	1	1
Gnaphosidae	*Micariaformicaria* (Sundevall, 1831)	24	10	7
Gnaphosidae	*Micariafulgens* (Walckenaer, 1802)	1	1	1
Gnaphosidae	*Micarianivosa* L. Koch, 1866	1	1	1
Gnaphosidae	*Micariapulicaria* (Sundevall, 1831)	17	12	11
Gnaphosidae	*Micariasilesiaca* L. Koch, 1875	7	2	3
Gnaphosidae	*Micariasubopaca* Westring, 1861	2	1	2
Gnaphosidae	*Scotophaeusscutulatus* (L. Koch, 1866)	1	1	1
Gnaphosidae	*Zelotesazsheganovae* Esyunin & Efimik, 1992	69	10	10
Gnaphosidae	*Zelotesclivicola* (L. Koch, 1870)	128	5	4
Gnaphosidae	*Zeloteselectus* (C. L. Koch, 1839)	1	1	1
Gnaphosidae	*Zelotesexiguus* (Müller & Schenkel, 1895)	17	3	2
Gnaphosidae	*Zeloteslatreillei* (Simon, 1878)	88	16	19
Gnaphosidae	*Zeloteslongipes* (L. Koch, 1866)	141	5	5
Gnaphosidae	*Zelotesmundus* (Kulczyński, 1897)	1	1	1
Gnaphosidae	*Zelotespetrensis* (C. L. Koch, 1839)	86	6	5
Gnaphosidae	*Zelotespseudogallicus* Ponomarev, 2007	34	1	2
Gnaphosidae	*Zelotessubterraneus* (C. L. Koch, 1833)	314	14	10
Hahniidae	*Antisteaelegans* (Blackwall, 1841)	169	8	9
Hahniidae	*Cicurinacicur* (Fabricius, 1793)	4	4	4
Hahniidae	*Hahnianava* (Blackwall, 1841)	20	5	5
Hahniidae	*Hahniaononidum* Simon, 1875	156	12	11
Hahniidae	*Hahniapusilla* C. L. Koch, 1841	95	6	8
Hahniidae	*Hahniasibirica* Marusik, Hippa & Koponen, 1996	4	1	1
Hahniidae	*Mastigusaarietina* (Thorell, 1871)	7	3	4
Linyphiidae	*Abacoproecessaltuum* (L. Koch, 1872)	276	16	12
Linyphiidae	*Agynetaaffinis* (Kulczyński, 1898)	85	9	10
Linyphiidae	*Agynetacauta* (O. Pickard-Cambridge, 1903)	5	3	3
Linyphiidae	*Agynetaconigera* (O. Pickard-Cambridge, 1863)	73	2	4
Linyphiidae	*Agynetamollis* (O. Pickard-Cambridge, 1871)	3	2	2
Linyphiidae	*Agynetaolivacea* (Emerton, 1882)	80	1	2
Linyphiidae	*Agynetaramosa* Jackson, 1912	2	2	2
Linyphiidae	*Agynetarurestris* (C. L. Koch, 1836)	2	2	2
Linyphiidae	*Agynetasaaristoi* Tanasevitch, 2000	9	4	5
Linyphiidae	*Agynetasubtilis* (O. Pickard-Cambridge, 1863)	154	5	5
Linyphiidae	*Allomengeascopigera* (Grube, 1859)	54	6	6
Linyphiidae	*Allomengeavidua* (L. Koch, 1879)	12	3	4
Linyphiidae	*Anguliphantesangulipalpis* (Westring, 1851)	81	11	9
Linyphiidae	*Araeoncuscrassiceps* (Westring, 1861)	1	1	1
Linyphiidae	*Asthenarguspaganus* (Simon, 1884)	2	2	2
Linyphiidae	*Baryphymatrifrons* (O. Pickard-Cambridge, 1863)	1	1	1
Linyphiidae	*Bathyphantesapproximatus* (O. Pickard-Cambridge, 1871)	2	2	2
Linyphiidae	*Bathyphantesgracilis* (Blackwall, 1841)	111	5	4
Linyphiidae	*Bathyphantesnigrinus* (Westring, 1851)	54	6	7
Linyphiidae	*Bathyphantesparvulus* (Westring, 1851)	5	3	4
Linyphiidae	*Bolyphantesalticeps* (Sundevall, 1833)	28	12	11
Linyphiidae	*Centromeritabicolor* (Blackwall, 1833)	10	2	5
Linyphiidae	*Centromerusarcanus* (O. Pickard-Cambridge, 1873)	15	3	4
Linyphiidae	*Centromerusbrevipalpus* (Menge, 1866)	14	1	2
Linyphiidae	*Centromerusclarus* (L. Koch, 1879)	11	1	2
Linyphiidae	*Centromerusincilium* (L. Koch, 1881)	24	2	1
Linyphiidae	*Centromerussylvaticus* (Blackwall, 1841)	63	8	14
Linyphiidae	*Ceratinellabrevipes* (Westring, 1851)	12	3	4
Linyphiidae	*Ceratinellabrevis* (Wider, 1834)	37	7	8
Linyphiidae	*Cnephalocotesobscurus* (Blackwall, 1834)	8	4	2
Linyphiidae	*Diplocephalusconnatus* Bertkau, 1889	1	1	1
Linyphiidae	*Diplocephalusdentatus* Tullgren, 1955	6	1	1
Linyphiidae	*Diplocephaluslatifrons* (O. Pickard-Cambridge, 1863)	18	2	2
Linyphiidae	*Diplocephaluspicinus* (Blackwall, 1841)	81	11	10
Linyphiidae	*Diplostylaconcolor* (Wider, 1834)	243	12	16
Linyphiidae	*Dismodicusbifrons* (Blackwall, 1841)	10	4	5
Linyphiidae	*Drapetiscasocialis* (Sundevall, 1833)	8	5	6
Linyphiidae	*Entelecaraacuminata* (Wider, 1834)	4	2	3
Linyphiidae	*Entelecaracongenera* (O. Pickard-Cambridge, 1879)	2	2	2
Linyphiidae	*Erigoneatra* Blackwall, 1833	1	1	1
Linyphiidae	*Erigonedentipalpis* (Wider, 1834)	12	6	5
Linyphiidae	*Erigonellahiemalis* (Blackwall, 1841)	5	3	2
Linyphiidae	*Erigonellaignobilis* (O. Pickard-Cambridge, 1871)	3	2	3
Linyphiidae	*Floroniabucculenta* (Clerck, 1758)	5	4	3
Linyphiidae	*Glyphesiscottonae* (La Touche, 1946)	1	1	1
Linyphiidae	*Glyphesisnemoralis* Esyunin & Efimik, 1994	10	2	2
Linyphiidae	*Gnathonariumdentatum* (Wider, 1834)	2	2	2
Linyphiidae	*Gongylidiellumlatebricola* (O. Pickard-Cambridge, 1871)	1	1	1
Linyphiidae	*Gongylidiellummurcidum* Simon, 1884	5	2	2
Linyphiidae	*Gongylidiumrufipes* (Linnaeus, 1758)	43	5	5
Linyphiidae	*Helophorainsignis* (Blackwall, 1841)	131	15	10
Linyphiidae	*Hylyphantesgraminicola* (Sundevall, 1830)	1	1	1
Linyphiidae	*Hypommabituberculatum* (Wider, 1834)	4	2	4
Linyphiidae	*Hypommafulvum* (Bösenberg, 1902)	2	1	1
Linyphiidae	*Hypselistesjacksoni* (O. Pickard-Cambridge, 1903)	2	1	1
Linyphiidae	*Incestophantescrucifer* (Menge, 1866)	3	2	2
Linyphiidae	*Kaestneriapullata* (O. Pickard-Cambridge, 1863)	7	5	5
Linyphiidae	*Lepthyphantesleprosus* (Ohlert, 1865)	2	2	2
Linyphiidae	*Linyphiahortensis* Sundevall, 1830	5	2	3
Linyphiidae	*Linyphiatenuipalpis* Simon, 1884	11	4	2
Linyphiidae	*Linyphiatriangularis* (Clerck, 1758)	174	24	22
Linyphiidae	*Lophommapunctatum* (Blackwall, 1841)	1	1	1
Linyphiidae	*Macrarguscarpenteri* (O. Pickard-Cambridge, 1895)	11	2	1
Linyphiidae	*Macrargusrufus* (Wider, 1834)	55	2	3
Linyphiidae	*Maropansibiricus* Tanasevitch, 2006	5	3	2
Linyphiidae	*Masosundevalli* (Westring, 1851)	16	6	7
Linyphiidae	*Megalepthyphantesnebulosus* (Sundevall, 1830)	1	1	1
Linyphiidae	*Megalepthyphantespseudocollinus* Saaristo, 1997	21	5	6
Linyphiidae	*Metapanamomopskaestneri* (Wiehle, 1961)	31	1	1
Linyphiidae	*Micrargusherbigradus* (Blackwall, 1854)	1	1	1
Linyphiidae	*Micrargussubaequalis* (Westring, 1851)	1	1	1
Linyphiidae	*Microlinyphiaimpigra* (O. Pickard-Cambridge, 1871)	1	1	1
Linyphiidae	*Microlinyphiapusilla* (Sundevall, 1830)	29	8	9
Linyphiidae	*Micronetaviaria* (Blackwall, 1841)	93	13	10
Linyphiidae	*Minyrioluspusillus* (Wider, 1834)	59	7	4
Linyphiidae	*Moebeliapenicillata* (Westring, 1851)	1	1	1
Linyphiidae	*Nerieneclathrata* (Sundevall, 1830)	20	12	9
Linyphiidae	*Nerieneemphana* (Walckenaer, 1841)	71	18	12
Linyphiidae	*Nerienemontana* (Clerck, 1758)	73	17	14
Linyphiidae	*Nerienepeltata* (Wider, 1834)	6	6	5
Linyphiidae	*Nerieneradiata* (Walckenaer, 1841)	34	10	8
Linyphiidae	*Obscuriphantesobscurus* (Blackwall, 1841)	1	1	1
Linyphiidae	*Oedothoraxapicatus* (Blackwall, 1850)	9	3	4
Linyphiidae	*Oedothoraxgibbosus* (Blackwall, 1841)	11	4	5
Linyphiidae	*Oedothoraxretusus* (Westring, 1851)	3	3	3
Linyphiidae	*Oryphantesangulatus* (O. Pickard-Cambridge, 1881)	1	1	1
Linyphiidae	*Osteariusmelanopygius* (O. Pickard-Cambridge, 1880)	1	1	1
Linyphiidae	*Palliduphantesalutacius* (Simon, 1884)	54	8	7
Linyphiidae	*Panamomopsmengei* Simon, 1926	15	6	7
Linyphiidae	*Pelecopsismengei* (Simon, 1884)	4	3	3
Linyphiidae	*Peponocraniumpraeceps* Miller, 1943	1	1	1
Linyphiidae	*Pityohyphantesphrygianus* (C. L. Koch, 1836)	6	3	4
Linyphiidae	*Pocadicnemispumila* (Blackwall, 1841)	25	6	7
Linyphiidae	*Porrhommaconvexum* (Westring, 1851)	8	2	2
Linyphiidae	*Porrhommamicrophthalmum* (O. Pickard-Cambridge, 1871)	2	2	2
Linyphiidae	*Porrhommapygmaeum* (Blackwall, 1834)	21	8	8
Linyphiidae	*Praestigiakulczynskii* Eskov, 1979	2	1	1
Linyphiidae	*Saaristoaabnormis* (Blackwall, 1841)	1	1	1
Linyphiidae	*Sauronrayi* (Simon, 1881)	1	1	1
Linyphiidae	*Silometopuselegans* (O. Pickard-Cambridge, 1873)	4	3	2
Linyphiidae	*Silometopusreussi* (Thorell, 1871)	1	1	1
Linyphiidae	*Stemonyphantesconspersus* (L. Koch, 1879)	1	1	1
Linyphiidae	*Stemonyphanteslineatus* (Linnaeus, 1758)	9	5	6
Linyphiidae	*Tallusiaexperta* (O. Pickard-Cambridge, 1871)	2	1	2
Linyphiidae	*Tapinocybaaffinis* Lessert, 1907	4	1	1
Linyphiidae	*Tapinocybabiscissa* (O. Pickard-Cambridge, 1873)	23	1	2
Linyphiidae	*Tapinocybainsecta* (L. Koch, 1869)	12	6	5
Linyphiidae	*Tapinopalongidens* (Wider, 1834)	10	7	4
Linyphiidae	*Tenuiphantesalacris* (Blackwall, 1853)	2	2	2
Linyphiidae	*Tenuiphantescristatus* (Menge, 1866)	2	1	1
Linyphiidae	*Tenuiphantesmengei* (Kulczyński, 1887)	74	11	10
Linyphiidae	*Tenuiphantesnigriventris* (L. Koch, 1879)	36	11	6
Linyphiidae	*Tenuiphantestenebricola* (Wider, 1834)	260	8	6
Linyphiidae	*Thyreostheniusparasiticus* (Westring, 1851)	9	2	2
Linyphiidae	*Tibioplusdiversus* (L. Koch, 1879)	8	2	2
Linyphiidae	*Trematocephaluscristatus* (Wider, 1834)	9	6	7
Linyphiidae	*Trichoncusaffinis* Kulczyński, 1894	19	1	1
Linyphiidae	*Trichoncusvasconicus* Denis, 1944	16	1	1
Linyphiidae	*Trichopternacito* (O. Pickard-Cambridge, 1873)	2	1	1
Linyphiidae	*Troxochrotascabra* Kulczyński, 1894	6	3	2
Linyphiidae	*Troxochrusscabriculus* (Westring, 1851)	19	3	3
Linyphiidae	*Walckenaeriaalticeps* (Denis, 1952)	40	4	5
Linyphiidae	*Walckenaeriaantica* (Wider, 1834)	34	4	4
Linyphiidae	*Walckenaeriaatrotibialis* (O. Pickard-Cambridge, 1878)	115	9	7
Linyphiidae	*Walckenaeriacucullata* (C. L. Koch, 1836)	34	3	3
Linyphiidae	*Walckenaeriadysderoides* (Wider, 1834)	3	2	2
Linyphiidae	*Walckenaeriafurcillata* (Menge, 1869)	5	1	2
Linyphiidae	*Walckenaerialepida* (Kulczyński, 1885)	1	1	1
Linyphiidae	*Walckenaeriamitrata* (Menge, 1868)	3	3	3
Linyphiidae	*Walckenaerianudipalpis* (Westring, 1851)	1	1	1
Linyphiidae	*Walckenaeriapicetorum* (Palmgren, 1976)	1	1	1
Linyphiidae	*Walckenaeriavigilax* (Blackwall, 1853)	2	2	2
Liocranidae	*Agroecabrunnea* (Blackwall, 1833)	53	7	6
Liocranidae	*Agroecacuprea* Menge, 1873	27	6	6
Liocranidae	*Agroecalusatica* (L. Koch, 1875)	13	5	6
Liocranidae	*Agroecamakarovae* Esyunin, 2008	2	1	1
Liocranidae	*Agroecaproxima* (O. Pickard-Cambridge, 1871)	163	10	9
Lycosidae	*Acantholycosalignaria* (Clerck, 1758)	12	5	4
Lycosidae	*Alopecosaaculeata* (Clerck, 1758)	673	8	6
Lycosidae	*Alopecosacuneata* (Clerck, 1758)	1307	15	16
Lycosidae	*Alopecosafabrilis* (Clerck, 1758)	3	2	2
Lycosidae	*Alopecosafarinosa* (Herman, 1879)	105	3	4
Lycosidae	*Alopecosainquilina* (Clerck, 1758)	6	5	3
Lycosidae	*Alopecosapulverulenta* (Clerck, 1758)	336	12	16
Lycosidae	*Alopecosasolitaria* (Herman, 1879)	63	2	2
Lycosidae	*Alopecosasulzeri* (Pavesi, 1873)	3	3	2
Lycosidae	*Alopecosataeniata* (C. L. Koch, 1835)	18	3	3
Lycosidae	*Arctosacinerea* (Fabricius, 1777)	4	2	1
Lycosidae	*Arctosafigurata* (Simon, 1876)	35	3	4
Lycosidae	*Arctosaleopardus* (Sundevall, 1833)	17	5	4
Lycosidae	*Arctosalutetiana* (Simon, 1876)	3	2	2
Lycosidae	*Arctosastigmosa* (Thorell, 1875)	11	4	4
Lycosidae	*Hygrolycosarubrofasciata* (Ohlert, 1865)	2	2	1
Lycosidae	*Lycosasingoriensis* (Laxmann, 1770)	1	1	1
Lycosidae	*Mustelicosadimidiata* (Thorell, 1875)	138	2	2
Lycosidae	*Pardosaagrestis* (Westring, 1861)	116	16	13
Lycosidae	*Pardosaagricola* (Thorell, 1856)	41	7	6
Lycosidae	*Pardosaalacris* (C. L. Koch, 1833)	32	5	4
Lycosidae	*Pardosaamentata* (Clerck, 1758)	83	8	11
Lycosidae	*Pardosafulvipes* (Collett, 1876)	2425	26	22
Lycosidae	*Pardosalugubris* (Walckenaer, 1802)	970	29	23
Lycosidae	*Pardosamaisa* Hippa & Mannila, 1982	43	3	2
Lycosidae	*Pardosapaludicola* (Clerck, 1758)	77	19	13
Lycosidae	*Pardosapalustris* (Linnaeus, 1758)	2430	23	20
Lycosidae	*Pardosaplumipes* (Thorell, 1875)	40	11	9
Lycosidae	*Pardosaprativaga* (L. Koch, 1870)	214	13	12
Lycosidae	*Pardosapullata* (Clerck, 1758)	1	1	1
Lycosidae	*Pardosaschenkeli* Lessert, 1904	3	1	1
Lycosidae	*Pardosasphagnicola* (Dahl, 1908)	124	16	12
Lycosidae	*Piratapiraticus* (Clerck, 1758)	60	13	9
Lycosidae	*Piratapiscatorius* (Clerck, 1758)	9	6	5
Lycosidae	*Piratatenuitarsis* Simon, 1876	4	2	2
Lycosidae	*Piratulahygrophila* (Thorell, 1872)	260	16	17
Lycosidae	*Piratulainsularis* (Emerton, 1885)	2	1	1
Lycosidae	*Trochosaruricola* (De Geer, 1778)	829	22	23
Lycosidae	*Trochosaspinipalpis* (F. O. Pickard-Cambridge, 1895)	21	10	11
Lycosidae	*Trochosaterricola* Thorell, 1856	343	18	12
Lycosidae	*Xerolycosaminiata* (C. L. Koch, 1834)	387	18	14
Lycosidae	*Xerolycosanemoralis* (Westring, 1861)	260	14	14
Mimetidae	*Erofurcata* (Villers, 1789)	8	5	4
Miturgidae	*Zoranemoralis* (Blackwall, 1861)	57	5	6
Miturgidae	*Zoraspinimana* (Sundevall, 1833)	40	13	8
Oxyopidae	*Oxyopesramosus* (Martini & Goeze, 1778)	10	6	4
Philodromidae	*Philodromusaureolus* (Clerck, 1758)	1	1	1
Philodromidae	*Philodromuscespitum* (Walckenaer, 1802)	149	18	18
Philodromidae	*Philodromusemarginatus* (Schrank, 1803)	5	5	5
Philodromidae	*Philodromusfuscomarginatus* (De Geer, 1778)	1	1	1
Philodromidae	*Philodromusmargaritatus* (Clerck, 1758)	3	2	1
Philodromidae	*Philodromuspoecilus* (Thorell, 1872)	11	7	5
Philodromidae	*Rhysodromushistrio* (Latreille, 1819)	22	4	4
Philodromidae	*Thanatusarenarius* L. Koch, 1872	212	6	4
Philodromidae	*Thanatusformicinus* (Clerck, 1758)	16	6	5
Philodromidae	*Thanatussabulosus* (Menge, 1875)	22	8	8
Philodromidae	*Thanatusstriatus* C. L. Koch, 1845	7	5	6
Philodromidae	*Tibellusmaritimus* (Menge, 1875)	24	10	11
Philodromidae	*Tibellusoblongus* (Walckenaer, 1802)	129	26	20
Pholcidae	*Pholcusalticeps* Spassky, 1932	3	3	2
Phrurolithidae	*Phrurolithusfestivus* (C. L. Koch, 1835)	78	15	15
Pisauridae	*Dolomedesfimbriatus* (Clerck, 1758)	24	11	7
Pisauridae	*Dolomedesplantarius* (Clerck, 1758)	47	9	11
Pisauridae	*Pisauramirabilis* (Clerck, 1758)	17	10	7
Salticidae	*Aelurillusv-insignitus* (Clerck, 1758)	2	1	1
Salticidae	*Attulusdzieduszyckii* (L. Koch, 1870)	38	8	4
Salticidae	*Attulusfloricola* (C. L. Koch, 1837)	22	9	7
Salticidae	*Attulussaltator* (O. Pickard-Cambridge, 1868)	5	3	2
Salticidae	*Attulusterebratus* (Clerck, 1758)	11	6	5
Salticidae	*Balluschalybeius* (Walckenaer, 1802)	14	11	11
Salticidae	*Dendryphantesrudis* (Sundevall, 1833)	15	2	6
Salticidae	*Euophrysfrontalis* (Walckenaer, 1802)	1	1	1
Salticidae	*Evarchaarcuata* (Clerck, 1758)	266	33	22
Salticidae	*Evarchafalcata* (Clerck, 1758)	95	23	17
Salticidae	*Heliophanusauratus* C. L. Koch, 1835	53	11	12
Salticidae	*Heliophanuscamtschadalicus* Kulczyński, 1885	1	1	1
Salticidae	*Heliophanuscupreus* (Walckenaer, 1802)	7	4	4
Salticidae	*Heliophanusdubius* C. L. Koch, 1835	5	3	4
Salticidae	*Heliophanusflavipes* (Hahn, 1832)	19	6	5
Salticidae	*Heliophanuslineiventris* Simon, 1868	2	1	1
Salticidae	*Marpissamuscosa* (Clerck, 1758)	1	1	1
Salticidae	*Marpissapomatia* (Walckenaer, 1802)	5	4	4
Salticidae	*Marpissaradiata* (Grube, 1859)	7	2	2
Salticidae	*Neonreticulatus* (Blackwall, 1853)	6	3	3
Salticidae	*Phlegrafasciata* (Hahn, 1826)	19	7	7
Salticidae	*Pseudeuophryserratica* (Walckenaer, 1826)	1	1	1
Salticidae	*Pseudiciusencarpatus* (Walckenaer, 1802)	4	1	1
Salticidae	*Salticuscingulatus* (Panzer, 1797)	8	3	3
Salticidae	*Sibianoraurocinctus* (Ohlert, 1865)	1	1	1
Salticidae	*Sibianorlarae* Logunov, 2001	2	2	2
Salticidae	*Sibianortantulus* (Simon, 1868)	1	1	1
Salticidae	*Synagelesvenator* (Lucas, 1836)	3	2	3
Salticidae	*Talaveraaequipes* (O. Pickard-Cambridge, 1871)	8	4	4
Salticidae	*Talaveraaperta* (Miller, 1971)	3	2	2
Salticidae	*Talaverapetrensis* (C. L. Koch, 1837)	1	1	1
Sparassidae	*Micrommatavirescens* (Clerck, 1758)	79	14	18
Tetragnathidae	*Metellinamengei* (Blackwall, 1869)	8	5	5
Tetragnathidae	*Metellinamerianae* (Scopoli, 1763)	1	1	1
Tetragnathidae	*Metellinasegmentata* (Clerck, 1758)	108	14	18
Tetragnathidae	*Pachygnathaclercki* Sundevall, 1823	14	7	7
Tetragnathidae	*Pachygnathadegeeri* Sundevall, 1830	175	7	11
Tetragnathidae	*Pachygnathalisteri* Sundevall, 1830	212	14	12
Tetragnathidae	*Tetragnathadearmata* Thorell, 1873	46	12	8
Tetragnathidae	*Tetragnathaextensa* (Linnaeus, 1758)	238	20	18
Tetragnathidae	*Tetragnathamontana* Simon, 1874	17	8	7
Tetragnathidae	*Tetragnathanigrita* Lendl, 1886	17	4	5
Tetragnathidae	*Tetragnathaobtusa* C. L. Koch, 1837	12	9	8
Tetragnathidae	*Tetragnathapinicola* L. Koch, 1870	134	19	17
Tetragnathidae	*Tetragnathashoshone* Levi, 1981	11	2	2
Tetragnathidae	*Tetragnathastriata* L. Koch, 1862	14	3	2
Theridiidae	*Asagenaphalerata* (Panzer, 1801)	25	5	6
Theridiidae	*Crustulinaguttata* (Wider, 1834)	53	10	10
Theridiidae	*Crustulinasticta* (O. Pickard-Cambridge, 1861)	2	2	2
Theridiidae	*Cryptachaeariparia* (Blackwall, 1834)	2	2	2
Theridiidae	*Dipoenatorva* (Thorell, 1875)	1	1	1
Theridiidae	*Enoplognathaovata* (Clerck, 1758)	54	15	15
Theridiidae	*Episinusangulatus* (Blackwall, 1836)	5	4	4
Theridiidae	*Episinustruncatus* Latreille, 1809	9	7	7
Theridiidae	*Euryopisflavomaculata* (C. L. Koch, 1836)	78	13	10
Theridiidae	*Euryopissaukea* Levi, 1951	5	1	3
Theridiidae	*Lasaeolaprona* (Menge, 1868)	1	1	1
Theridiidae	*Lasaeolatristis* (Hahn, 1833)	5	5	5
Theridiidae	*Neottiurabimaculata* (Linnaeus, 1767)	33	14	14
Theridiidae	*Parasteatodalunata* (Clerck, 1758)	8	3	4
Theridiidae	*Parasteatodasimulans* (Thorell, 1875)	5	3	3
Theridiidae	*Parasteatodatabulata* (Levi, 1980)	43	8	7
Theridiidae	*Phyllonetaimpressa* (L. Koch, 1881)	136	23	17
Theridiidae	*Phyllonetasisyphia* (Clerck, 1758)	18	10	7
Theridiidae	*Platnickinatincta* (Walckenaer, 1802)	2	1	2
Theridiidae	*Robertusarundineti* (O. Pickard-Cambridge, 1871)	17	9	10
Theridiidae	*Robertuslividus* (Blackwall, 1836)	57	10	9
Theridiidae	*Robertusneglectus* (O. Pickard-Cambridge, 1871)	4	3	3
Theridiidae	*Robertusscoticus* Jackson, 1914	1	1	1
Theridiidae	*Simitidionsimile* (C. L. Koch, 1836)	4	2	2
Theridiidae	*Steatodaalbomaculata* (De Geer, 1778)	8	2	1
Theridiidae	*Steatodabipunctata* (Linnaeus, 1758)	88	14	11
Theridiidae	*Steatodacastanea* (Clerck, 1758)	27	12	8
Theridiidae	*Steatodagrossa* (C. L. Koch, 1838)	46	7	5
Theridiidae	*Theridioninnocuum* Thorell, 1875	7	1	2
Theridiidae	*Theridionpictum* (Walckenaer, 1802)	55	9	11
Theridiidae	*Theridionpinastri* L. Koch, 1872	5	2	3
Theridiidae	*Theridionvarians* Hahn, 1833	49	14	13
Thomisidae	*Coriarachnedepressa* (C. L. Koch, 1837)	6	3	1
Thomisidae	*Ebrechtellatricuspidata* (Fabricius, 1775)	161	20	19
Thomisidae	*Misumenavatia* (Clerck, 1758)	127	21	18
Thomisidae	*Ozyptilaclaveata* (Walckenaer, 1837)	29	3	4
Thomisidae	*Ozyptilapraticola* (C. L. Koch, 1837)	359	19	16
Thomisidae	*Ozyptilatrux* (Blackwall, 1846)	72	10	14
Thomisidae	*Psammitissabulosus* (Hahn, 1832)	1	1	1
Thomisidae	*Spiracmestriatipes* (L. Koch, 1870)	164	12	8
Thomisidae	*Tmaruspiger* (Walckenaer, 1802)	32	12	7
Thomisidae	*Xysticusaudax* (Schrank, 1803)	9	4	3
Thomisidae	*Xysticusbifasciatus* C. L. Koch, 1837	41	7	9
Thomisidae	*Xysticuscristatus* (Clerck, 1758)	80	20	17
Thomisidae	*Xysticuskochi* Thorell, 1872	16	7	10
Thomisidae	*Xysticuslanio* C. L. Koch, 1835	5	4	4
Thomisidae	*Xysticuslineatus* (Westring, 1851)	1	1	1
Thomisidae	*Xysticusluctator* L. Koch, 1870	160	8	9
Thomisidae	*Xysticusluctuosus* (Blackwall, 1836)	44	6	6
Thomisidae	*Xysticusulmi* (Hahn, 1831)	111	29	21
Titanoecidae	*Titanoecapraefica* (Simon, 1870)	2	1	1
Titanoecidae	*Titanoecaquadriguttata* (Hahn, 1833)	11	2	2
Titanoecidae	*Titanoecaschineri* L. Koch, 1872	12	3	3
Titanoecidae	*Titanoecaspominima* (Taczanowski, 1866)	43	3	3
Uloboridae	*Uloboruswalckenaerius* Latreille, 1806	2	2	2
